# Advanced porous hip implants: A comprehensive review

**DOI:** 10.1016/j.heliyon.2024.e37818

**Published:** 2024-09-14

**Authors:** Babak Ziaie, Xavier Velay, Waqas Saleem

**Affiliations:** a Department of Mechanical and Manufacturing Engineering, Atlantic Technological University, Ash Lane, Sligo, F91 YW50, Ireland; b Centre for Precision Engineering Material and Manufacturing Research (PEM Research Centre), Atlantic Technological University, Ash Lane, Sligo, F91 YW50, Ireland; c Centre for Mathematical Modelling and Intelligent Systems for Health and Environment (MISHE), Atlantic Technological University, Ash Lane, F91 YW50, Sligo, Ireland; d School of Mechanical Engineering, Technological University Dublin, Dublin, Ireland

**Keywords:** Bone morphology, Bone biomechanical parameters, Solid implants' complications, Porous structures, Porous implants

## Abstract

The field of orthopaedic implants has experienced significant advancements in recent years, transforming the approach to orthopaedic treatments. Amongst these advancements, porous structures have emerged as a promising solution to address the limitations of traditional solid implants. This comprehensive review paper offers a thorough overview of the importance of advanced porous hip implants, focusing on three key areas bone morphology and biomechanical parameters, complications associated with solid implants, and the benefits of porous structures and porous implants.

Understanding the intricate interplay between bone morphology and biomechanical parameters is crucial when designing orthopaedic implants. Mimicking the native bone structure ensures optimal osseointegration, load distribution, and long-term success. Porous implants closely resemble natural bone structures, facilitating improved integration and biomechanical compatibility.

Complications with solid implants are a significant concern in orthopaedic procedures. Stress shielding, cortical hypertrophy, and micromotion can lead to implant failure or revision surgeries. By contrast, porous structures promise to mitigate these issues by promoting bone ingrowth, reducing stress concentrations, and providing stability at the bone-implant interface.

The benefits of porous structures and porous implants go beyond addressing solid implant complications. These structures enhance bone in-growth potential, strengthening integration and long-term stability. The interconnected porosity promotes nutrient diffusion and new blood vessel formation, supporting healing and minimizing infection risk.

Furthermore, porous implants exhibit improved mechanical properties, such as lower elastic modulus and higher energy absorption, that better match those of bone. This feature helps alleviate stress shielding and enhances the overall performance and longevity of the implant.

In conclusion, advanced porous implants have tremendous potential in orthopaedics. By closely mimicking native bone structure and reducing complications associated with solid implants, they can revolutionize orthopaedic treatments. Further research and development are warranted to fully exploit the potential of these innovative solutions and improve patient outcomes.

## 1 Introduction

In contemporary society, biomedical implants have role been paramount in individuals' lives and the healthcare system. From a commercial perspective, most orthopaedic devices and prostheses are constructed using solid, dense isotropic materials, including Titanium alloys, Co-Cr alloys, and stainless-steel alloys (SS. 316L) [[1], [2], [3], [4]]. Notably, Ti-6Al-4V is the most extensively used metallic material within this domain, owing to its excellent biocompatibility, ductility, superior corrosion resistance, and lower elasticity modulus compared to steels [5,6]. Nevertheless, despite possessing a relatively lower elasticity modulus, titanium alloys still exhibit significantly higher values (five to ten times) than bone tissues. Consequently, this mechanical property mismatch can instigate bone resorption at the prosthesis-bone interface due to the well-known stress shielding effect, thereby augmenting the likelihood of implant failure through implant loosening or bone fracture [7,8]. The stress shielding effect represents a phenomenon in which a solid implant impedes the transmission of applied loads to the bone tissues [9]. Complementarily, Wolff's law posits that if a specific bone experiences heightened loading, it would undergo remodelling to enhance its strength and accommodate the applied loads [10].

Nevertheless, stress shielding engenders reduced bone density and weakened structure, ultimately culminating in implant failure [11]. Researchers have explored various strategies to alleviate or eliminate the stress shielding effect [12], with the most productive among them involving the utilisation of porous structures instead of solid, dense implants [13,14]. Cutting-edge technologies like additive manufacturing (AM) have revolutionized the production of intricate lattice cell structures, which hold tremendous promise in the realm of biomedical devices [15,16]. The elasticity modulus of these porous cells can be tailored by modifying key parameters, such as pore/strut size, porosity or relative density, and cell morphology. Notably, one practical approach introduces adjustable porosity or relative density, a concept pioneered by Gibson and Ashby, whose model remains widely recognized for its ability to predict the mechanical properties of cellular materials [[17], [18], [19]].

This review paper addresses the pivotal role advanced porous implants play in orthopaedic applications, focusing on hip joint implants, covering bone morphology and biomechanical parameters, complications associated with conventional solid implants, and the advantages of porous structures and implants. Despite extensive research on implant porous implants design evolution and manufacturing criteria, a thorough review encompassing all vital parameters in designing, modelling, and analysing porous implants in conjunction with host bones and necessary biological criteria is notably lacking. Our review aims to fill this gap by providing detailed step-by-step information on modelling and analysing implants, including bone material models, international standards, and recent methodologies. We established stringent inclusion criteria and conducted a thorough search, using specific keywords across prominent research databases, emphasizing literature from the past 15 years, particularly the last 5 years. By synthesising this comprehensive body of research, we aspire to significantly enhance the understanding and advancement of porous implant technology in orthopaedic applications. We offer valuable insights for researchers, clinicians, and stakeholders to promote improved patient outcomes and the continued evolution of orthopaedic implant technology.

## 2 Bone morphology and biomechanical parameters

When investigating bone morphology, researchers consider multiple scales to comprehensively understand its structure. These scales encompass sub-nano structure, nanostructure, microstructure, and macrostructure. At the sub-nano structure level, the focus is on the tropocollagen, a fundamental component of bone, with a diameter of approximately 1.5 nm. Moving up to the macrostructure level, cortical and cancellous bones are examined, which constitute the more significant, visible structures [20].

Careful consideration of the microstructure scale is crucial in designing bone implants, particularly for hip joint implants. This scale ranges from 300 to 800 μm and encompasses parameters necessary for successful cell seeding, bone ingrowth, and vascularization. These factors are vital for ensuring the implant's integration with the surrounding bone tissue and for promoting long-term stability and functionality [21,22].

Furthermore, the macrostructure scale becomes significant when investigating the mechanical behaviour of bone implants and for studying material properties models applicable to bones in analytical or numerical studies. By examining the macrostructure, researchers can gain insights into the implant's overall structural integrity, load-bearing capacity, and durability. This information aids in developing and refining implant designs to meet the specific mechanical requirements of the bone site [4,23]. Considering bone morphology at different scales allows for a more comprehensive analysis of its intricate structure and enables researchers to tailor implant designs to specific biological and mechanical demands. By understanding bone's sub-nano, nano, micro, and macro structures, advancements can be made in developing more effective and successful bone implants, particularly hip joint replacements.

The femur, known as the longest and most robust bone in the human body, boasts a length of approximately 45 mm, accounting for roughly a quarter of an individual's height. This bone articulates with the hip bone to form the hip joint at its upper end, while its lower end connects with the patella and tibia. In an upright stance, the femur effectively transfers the body weight from the hip bone to the tibia. Structurally, the femur comprises three distinct components: the proximal epiphysis (upper end), the diaphysis (shaft), and the distal epiphysis (lower back). Cortical (compact) and cancellous (spongy) bone are prevalent in most bone structures, however their distribution and concentration vary depending on the bone's specific functionality. Although cortical and cancellous bone share common matrix materials and cells, they exhibit contrasting organizational patterns. Cortical bone shows high density, rendering it resistant to compressive forces.

The porous three-dimensional latticework of cancellous bone provides a lightweight structure with optimal structural integrity.

**Fig. 1:**
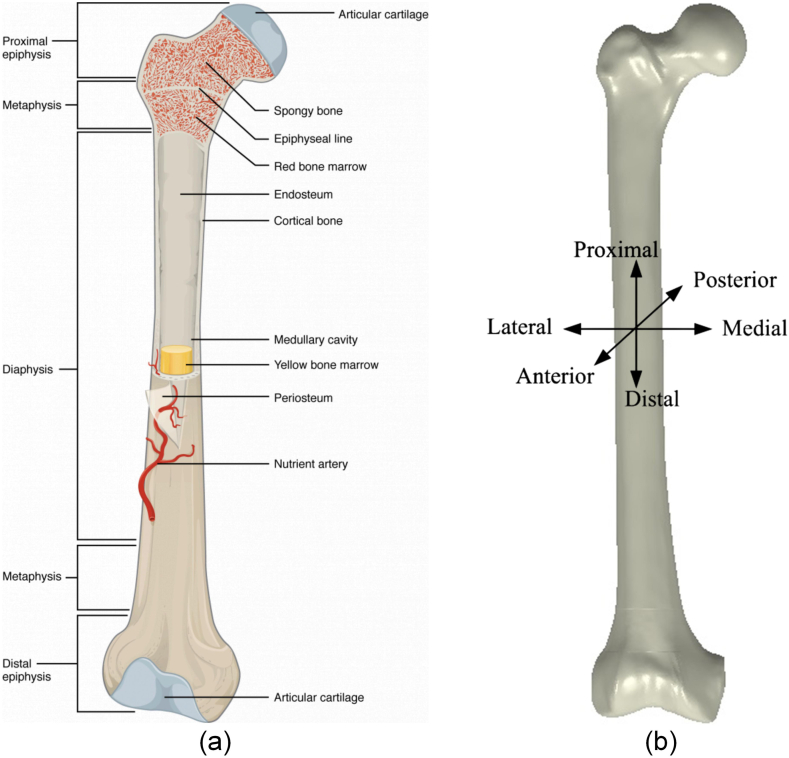
(a) Typical femur [24] (b) Right femur reference system.

**Fig. 2:**
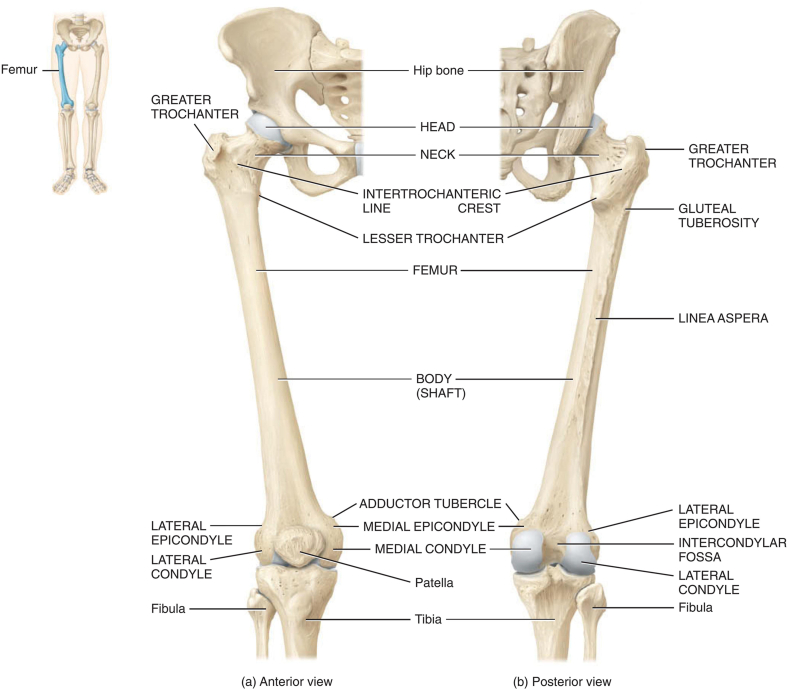
Right femur [25].

Furthermore, cancellous bone demonstrates remarkable adaptability through remodelling, allowing it to accommodate the body's changing physiological requirements. Fig. 1(a) [24] details the structure of a right femur bone, including the outer shell and the deeper layer. Fig. 1(b) illustrates the standard terms of location for the right femur anatomy. Fig. 2(a) & (b) [25], respectively, show the detailed anatomies of a femur from both the anterior and posterior views.

The stability of a hip joint implant is significantly influenced by the quality of the femur, particularly the proximal epiphysis. As a result, extensive research has investigated femur quality, cortical thickness, and classification methods. Amongst the various classification systems proposed, the Dorr classification, initially introduced by Dorr et al. (1993), has emerged as one of the most well-known and widely accepted schemes [26].

**Fig. 3:**
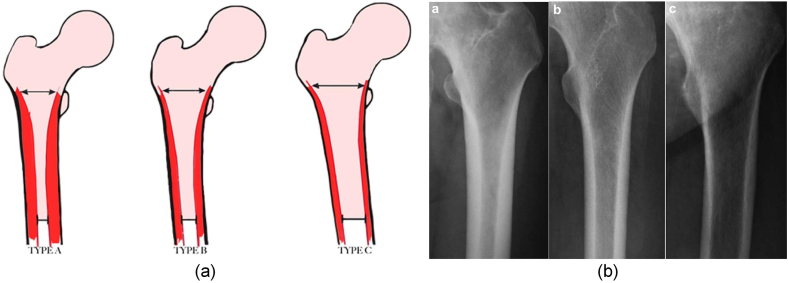
(a) Dorr classification [27] (b) Radiography of preoperative proximal femoral geometry categorized using the classification proposed by Dorr [28].

The Dorr classification system aims to categorize the proximal femur into distinct types, namely type A, type B, and type C, as depicted in Fig. 3(a), and involves the utilisation of two indices, namely the cortical thickness index and the calcar to canal ratio. The cortical thickness index measures the ratio between (i) the difference in diameter between the femoral diaphysis and the intramedullary canal (at a location 10 cm distal to the mid-lesser trochanter), and (ii) the diameter of the femoral diaphysis at the same point. On the other hand, the calcar-to-canal ratio quantifies the proportion of the intramedullary canal's isthmus (at a point 10 cm distal to the mid-lesser trochanter) to the diameter of the intramedullary canal at the calcar (located 3 cm distal to the mid-lesser trochanter). This classification is primarily based on radiographic assessment, utilising images such as those shown in Fig. 3(b) of patients who require total hip arthroplasty (THA). By employing this classification system, orthopaedic surgeons and clinicians can make informed decisions regarding the suitability of cementless implants for individual patients based on the quality of their femur [27,28].

Type A femurs are characterized by a thick cortical shell, indicating good bone quality and high density. This type of femur provides ample support for cementless implants, making them a suitable choice for THA. Type B femurs, on the other hand, exhibit a thinner cortical shell and a higher proportion of cancellous bone. These femurs require careful evaluation and consideration when choosing the appropriate implant type. Type C femurs, the least desirable for cementless implants, are characterized by a thin cortical shell and a significant presence of cancellous bone, indicating poor bone quality and density. Orthopaedic surgeons can determine the optimal surgical approach and implant selection by assessing the femoral bone quality and cortical thickness and implementing both qualitative and quantitative methods. Cementless implants are generally preferred for type A femurs, due to their favourable bone quality and density. In contrast, type B and type C femurs may necessitate alternative implant options, such as cemented or hybrid fixation, to ensure long-term stability and successful outcomes [[29], [30], [31], [32], [33]].

The quality of the femur, including its cortical thickness, is influenced by many factors, including age, gender, disease level, physiology, and lifestyle. As cortical thickness directly correlates with the stability of implants in THA, researchers have undertaken efforts to measure cortical bone thickness in the proximal femur. Various methods have been employed, such as Quantitative Computed Tomography (QCT), High Resolution peripheral Quantitative Computed Tomography (HRpQCT), Dual-energy X-ray Absorptiometry (DXA), Computed Tomography (CT), and Standard Radiography (XR). The diverse studies on this topic have provided valuable insights. Table 1 summarises these in terms of the number of samples, methods used, and the reported values for cortical thicknesses. These measurement techniques and reported values contribute to the overall understanding of femur quality and aid in optimising implant stability in THA procedures.

**Table 1:** Studies investigating cortical bone thickness.

**Author**	**Method**	**Sample**	**Section**	**Cortical thickness (mm)**
F. Schmidutz et al. (2021) [34]	XR & CT	54 Samples (19–90 years)	Humeral Diaphysis	4.05–9.95 (Mean: 7)
W. Du et al. (2018) [35]	CT & Mimics software	36 Females 59 Males (16–83 Years)	Femur	0.96–8.7 (Mean: 4.83)
G.M. Treece et al. (2015) [36]	QCT & HRpQCT	18 Females 17 Males (59–96 years)	Proximal femur	0.3–6 (Mean: 3.15)
Y. Long et al. (2015) [37]	QCT & DXA	210 samples (Mean age 66.5)	8 Sections of femur∗	1.41–5.39 (Mean∗: 3.57)
V.J. Cvetkovic et al. (2013) [38]	Review paper	–	Shaft	2.78–7.06 (Mean: 4.92)
K.E.S. Poole et al. (2011) [39]	Mapping using CT	65 Femals (Mean age 67.5)	Proximal femur	1–4 Thickness map
S.L. Croker et al. (2009) [40]	Video camera & digitizer	Data set	Femoral midshaft	Mean: 7.03

### 2.1 Mechanical properties of bone

The unique mechanical properties of bone stem from its composition of organic and inorganic components. Acknowledging that these material properties are subject to interpretation, based on factors such as age, overall health, and anatomical location within the body is crucial. Moreover, bone is a dynamic tissue that continuously undergoes remodelling, allowing it to adapt to mechanical demands and facilitate repair processes. From a mechanical standpoint, bone can be modelled as a complete isotropic or linear orthotropic (transversely isotropic) material. Generally, the relationship between stress and strain in bone falls within the linear region and can be expressed using a stiffness matrix. Hook's law can be formulated as Equation (1) [[41], [42], [43]] to express the general case:

(1) $$\sigma_{ij}=C_{ijkl}\varepsilon_{kl}$$

The fourth-order elasticity tensor, C_ijkl_, is a fundamental mathematical entity employed in continuum mechanics to describe the linear elastic behaviour of anisotropic materials. It establishes a linear relationship between the stress and strain tensors, characterising the material's response to mechanical deformation. The elasticity tensor is a 4th-order tensor with 81 components initially. However, exploiting its symmetry properties enables its reduction to a tensor with 21 independent components, as demonstrated in Equation (2).

(2) $$\left[\begin{array}{c} \begin{array}{c} \sigma_{11}\\ \sigma_{22}\\ \sigma_{33} \end{array}\\ \begin{array}{c} \sigma_{23}\\ \sigma_{13}\\ \sigma_{12} \end{array} \end{array}\right]=\left[\begin{array}{cc} \begin{array}{ccc} C_{1111} & C_{1122} & C_{1133}\\ & C_{2222} & C_{2233}\\ & & C_{3333} \end{array} & \begin{array}{ccc} C_{1123} & C_{1113} & C_{1112}\\ C_{2223} & C_{2213} & C_{2212}\\ C_{3323} & C_{3313} & C_{3312} \end{array}\\ sym\text{.} & \begin{array}{ccc} C_{2323} & C_{2313} & C_{2312}\\ & C_{1313} & C_{1312}\\ & & C_{1212} \end{array} \end{array}\right]\left[\begin{array}{c} \begin{array}{c} \varepsilon_{11}\\ \varepsilon_{22}\\ \varepsilon_{33} \end{array}\\ \begin{array}{c} \gamma_{23}=2\;\varepsilon_{23}\\ \gamma_{13}=2{\;\varepsilon }_{13}\\ \gamma_{12}=2\;\varepsilon_{12} \end{array} \end{array}\right]$$

The extensive use of the fourth-order elasticity tensor lies in its application to studying anisotropic materials, which exhibit varying mechanical properties in different directions. By incorporating the elasticity tensor, researchers can gain valuable insights into the behaviour and response of anisotropic materials under mechanical loading conditions. This tensor plays a vital role in understanding and analysing the mechanical properties of materials, particularly in fields such as materials science and engineering.

In the case of transversely isotropic materials, the elasticity tensor can be simplified by exploiting the material's symmetry properties. This simplified form is particularly suitable for modelling materials such as cortical bone and specific biological tissues exhibiting transverse isotropy. Transverse isotropy refers to a material having an axis of symmetry, with its properties being the same in two orthogonal (transverse) directions perpendicular to the axis.

For transversely isotropic materials, the fourth-order elasticity tensor can be expressed in a reduced form, with five independent coefficients, as shown in Equation (3):

(3) $$C_{ijkl}=\left[\begin{array}{cc} \begin{array}{ccc} C_{1111} & C_{1122} & C_{1133}\\ & C_{1111} & C_{1133}\\ & & C_{3333} \end{array} & \begin{array}{ccc} 0\; & 0\; & 0\\ 0\; & 0\; & 0\\ 0\; & 0\; & 0 \end{array}\;\\ sym\text{.} & \begin{array}{ccc} C_{1313} & \;0 & 0\\ & C_{1313} & 0\\ & & \frac{C_{1111}-C_{1122}}{2} \end{array} \end{array}\right]$$

The relationship between stress and strain can be rewritten as Equation (4):

$$E_{1}=E_{2}\;\left(In-Plane\right),E_{3}\left(Transverse\right),\nu_{12}=\nu_{21},\nu_{31}=\nu_{32},\nu_{13}=\nu_{23},\frac{\nu_{31}}{E_{3}}=\frac{\nu_{13}}{E_{1}}=\frac{\nu_{23}}{E_{2}},G_{13}=G_{23},G_{12}=\frac{E_{1}}{{2(1+\nu }_{12})}=\frac{E_{2}}{{2(1+\nu }_{21})}$$

(4) $$\left[\begin{array}{c} \begin{array}{c} \varepsilon_{11}\\ \varepsilon_{22}\\ \varepsilon_{33} \end{array}\\ \begin{array}{c} \gamma_{23}=2\;\varepsilon_{23}\\ \gamma_{13}=2{\;\varepsilon }_{13}\\ \gamma_{12}=2\;\varepsilon_{12} \end{array} \end{array}\right]=\left[\begin{array}{cc} \begin{array}{ccc} \frac{1}{E_{1}} & -\frac{\nu_{12}}{E_{1}} & -\frac{\nu_{31}}{E_{3}}\\ -\frac{\nu_{12}}{E_{1}} & \frac{1}{E_{1}} & -\frac{\nu_{31}}{E_{3}}\\ -\frac{\nu_{31}}{E_{3}} & -\frac{\nu_{31}}{E_{3}} & \frac{1}{E_{3}} \end{array} & \begin{array}{ccc} 0 & 0 & 0\\ 0 & 0 & 0\\ 0 & 0 & 0 \end{array}\\ \begin{array}{ccc} 0 & 0 & 0\\ 0 & 0 & 0\\ 0 & 0 & 0 \end{array} & \begin{array}{ccc} \frac{1}{G_{23}} & 0 & 0\\ 0 & \frac{1}{G_{23}} & 0\\ 0 & 0 & \frac{{2(1+\nu }_{12})}{E_{1}} \end{array} \end{array}\right]\left[\begin{array}{c} \begin{array}{c} \sigma_{11}\\ \sigma_{22}\\ \sigma_{33} \end{array}\\ \begin{array}{c} \sigma_{23}\\ \sigma_{13}\\ \sigma_{12} \end{array} \end{array}\right]$$

Equation (4) relates the components of strain to the components of stress through the compliance tensor S_ijkl_, which is the inverse of the elasticity tensor.

When bones are treated as isotropic materials, the stress-strain relationship can be further simplified, using only two independent coefficients. The elasticity tensor for isotropic bones can be expressed as Equation (5):

(5) $$C_{ijkl}=\left[\begin{array}{cc} \begin{array}{ccc} C_{1111} & C_{1122} & C_{1133}\\ & C_{1111} & C_{1133}\\ & & C_{3333} \end{array} & \begin{array}{ccc} 0\; & 0\; & 0\\ 0\; & 0\; & 0\\ 0\; & 0\; & 0 \end{array}\;\\ sym\text{.} & \begin{array}{ccc} C_{1313} & \;0 & 0\\ & C_{1313} & 0\\ & & \frac{C_{1111}-C_{1122}}{2} \end{array} \end{array}\right]$$

This equation represents the reduced form of the elasticity tensor for isotropic bones, where C_ijkl_ represents the tensor components based on the bone material's particular properties. This simplified form assumes that the bone material exhibits the same mechanical properties in all directions.

The relationship between stress and strain for isotropic bones can be expressed using the compliance tensor, as shown in Equation (6):

(6) $$\varepsilon_{ij}=\frac{1}{2\mu }\sigma_{ij}-\frac{\lambda }{2\mu \left(2\mu +3\lambda \right)}\sigma_{kk}\delta_{ij}$$

In this equation, ε_ij_ represents the components of strain, σ_ij_ represents the components of stress, μ represents the shear modulus, λ represents Lamé's first parameter, and δ_ij_ represents the Kronecker delta.

Furthermore, the stress and strain can be related using the compliance tensor by considering the following equation:

(7) $$\left[\begin{array}{c} \begin{array}{c} \varepsilon_{11}\\ \varepsilon_{22}\\ \varepsilon_{33} \end{array}\\ \begin{array}{c} \gamma_{23}=2\;\varepsilon_{23}\\ \gamma_{13}=2{\;\varepsilon }_{13}\\ \gamma_{12}=2\;\varepsilon_{12} \end{array} \end{array}\right]=\left[\begin{array}{cc} \begin{array}{ccc} \frac{1}{E} & -\frac{\nu }{E} & -\frac{\nu }{E}\\ -\frac{\nu }{E} & \frac{1}{E} & -\frac{\nu }{E}\\ -\frac{\nu }{E} & -\frac{\nu }{E} & \frac{1}{E} \end{array} & \begin{array}{ccc} 0 & 0 & 0\\ 0 & 0 & 0\\ 0 & 0 & 0 \end{array}\\ \begin{array}{ccc} 0 & 0 & 0\\ 0 & 0 & 0\\ 0 & 0 & 0 \end{array} & \begin{array}{ccc} \frac{1}{G} & 0 & 0\\ 0 & \frac{1}{G} & 0\\ 0 & 0 & \frac{1}{G} \end{array} \end{array}\right]\left[\begin{array}{c} \begin{array}{c} \sigma_{11}\\ \sigma_{22}\\ \sigma_{33} \end{array}\\ \begin{array}{c} \sigma_{23}\\ \sigma_{13}\\ \sigma_{12} \end{array} \end{array}\right]$$

In Equation (7), E represents Young's modulus, ν represents Poisson's ratio, and G represents the shear modulus.

These equations provide a simplified approach to characterise the stress-strain relationship in isotropic bone materials, facilitating the analysis and modelling of bone behaviour under mechanical loading conditions.

In various research studies, investigators have employed either the complete isotropic model or the transversely isotropic model to characterise the mechanical properties of bones. Table 2, Table 3 present the reported parameters associated with the complete isotropic and transversely isotropic models, respectively, to provide a comprehensive overview of the implemented models.

**Table 2:** Mechanical properties of bones considered as complete isotropic.

**Author**	**Material**	**Elasticity Modulus (GPa)**	**Poisson's ratio**
L. Guo et al. (2022) [4]	Cortical bone	17–20	–
	Trabecular bone	0.2–2	–
X. Gao et al. (2022) [44]	Cortical bone	13.7	0.3
	Trabecular bone	1.3	0.3
G. Cortis et al. (2022) [45]	Cortical bone	18	0.4
	Trabecular bone	0.386	0.33
E. Yang et al. (2019) [46]	Cortical bone	15.9–18.7	–
	Trabecular bone	0.39	–
Z. Zhang et al. (2018) [47]	Cortical bone	12	0.3
	Trabecular bone	0.1	0.2
B. Dabrowski et al. (2010) [48]	Cortical bone	12.4–22	–
	Trabecular bone	0.1–2	–
K. Alvarez et al. (2009) [5]	Cortical bone	7.1–27.6	–
	Trabecular bone	0.1–10.4	–

**Table 3:** Mechanical properties of bones considered as transversely isotropic.

**Author**	**Bone**	**Elasticity Modulus (GPa)**	**Shear Modulus (GPa)**	**Poisson's ratio**	**Density (g/cm**^**3**^**)**
S. E. Alkhatib et al. (2019) [49,50]	Cortical	E_1_ = E_2_ = 11.5 E_3_ = 17	G_13_ = G_23_ = 3.3 G_12_ = 3.6	ν_12_ = ν_21_ = 0.51 ν_31_ = ν_32_ = 0.31	–
	Trabecular	0.4	–	0.3	–
Y. He et al. (2018) [51]	Cortical	E_1_ = E_2_ = 11.5 E_3_ = 17	G_13_ = G_23_ = 3.3 G_12_ = 3.6	ν_12_ = ν_21_ = 0.51 ν_13_ = ν_23_ = 0.31	1.8
	Trabecular	1	–	0.3	0.45
A. Calvo-E al. (2018) [52]	Cortical	E_1_ = E_2_ = 11.3 E_3_ = 22	G_13_ = G_23_ = 5.4 G_12_ = 3.8	ν_12_ = ν_21_ = 0.0484 ν_13_ = ν_23_ = 0.203	–
	Trabecular	E_1_ = E_2_ = 0.14 E_3_ = 0.2	G_13_ = G_23_ = 0.0483 G_12_ = 0.0483	ν_12_ = ν_21_ = 0.045 ν_13_ = ν_23_ = 0.315	–
X. Wang et al. (2016) [20]	Cortical	E_1_ = E_2_ = 10.1 ± 2.4 E_3_ = 17.9 ± 3.9	G_13_ = G_23_ = 3.3 ± 0.4 G_12_ = 3.3 ± 0.4	ν_12_ = ν_21_ = 0.4 ± 0.16 ν_13_ = ν_23_ = 0.62 ± 0.26	–
	Trabecular	0.441 ± 0.271	–	–	–
N. F Al Zoubi et al. (2022) [53]	Cortical	E_1_ = E_2_ = 11.5 E_3_ = 17	G_13_ = G_23_ = 3.3 G_12_ = 3.6	ν_12_ = ν_21_ = 0.51 ν_13_ = ν_23_ = 0.31	–
	Trabecular	0.4	–	0.3	–
B. Ziaie et al. (2021) [54]	Cortical	E_1_ = E_2_ = 12.6 E_3_ = 19.4	G_13_ = G_23_ = 5.7 G_12_ = 4.85	ν_12_ = ν_21_ = 0.3 ν_13_ = ν_23_ = 0.253	1.7
	Trabecular	E_1_ = E_2_ = 1.148 E_3_ = 0.27	G_13_ = G_23_ = 0.068 G_12_ = 0.434	ν_12_ = ν_21_ = 0.055 ν_13_ = ν_23_ = 0.322	0.27

It is worth mentioning that a study by H. Mehboob et al. (2020) [55] considered cortical bone as an anisotropic material. Additionally, an alternative approach to determining the material properties of bone involves calculating them based on the grayscale value (G) derived from CT data or the Hounsfield scale (HU), as outlined in Table 4 [13,[56], [57], [58]]. However, it is noteworthy that the results obtained using these material properties exhibit negligible disparities [57].

**Table 4:** Material properties of bones using CT data.

**Author**	**Elasticity modulus (Pa)**	**Density (gr/cm**^**3**^**)**
M. Rana et al. (2023) [59]	E = 6,850 × ρ^1.49^	ρ = 0.505167 + 0.0005412087 × HU
C. Sun et al. (2018) [56]	E = −388.8 + 5,925 × ρ	ρ = −13.4 + 1,017 × G
S. Arabnejad et al. (2016) [57]	E = 1,904 × ρ^1.64^, ρ < 0.95 E = 2,065 × ρ^3.09^, ρ > 0.95	0 to 1,567HU

## 3 Solid hip joint implants and their complications

Total hip arthroplasty is a highly dependable and cost-effective surgical procedure in orthopaedics. Its consistent success rate has made it a reliable treatment option for patients. However, the choice of techniques, preoperative considerations, surgical approaches, and implant types can vary based on several factors, including femur quality, gender, and physiological characteristics [[60], [61], [62]]International standards have been established for implant manufacturing for hip joint implants. These standards outline crucial considerations about materials, load conditions, implant specifications, and manufacturing requirements. For a comprehensive overview of these standards, refer to Table 5.

**Table 5:** Hip joint standards.

**Standard**	**Description**
ISO 7206-1-2008	Classification and designation of dimensions
ISO 7206-4-2010E	Determination of endurance properties and performance of stemmed femoral components (Loading Conditions)
ISO 5832–3:2021	Implants for surgery - Metallic materials - Part 3: Wrought titanium 6-aluminium 4-vanadium alloy
ASTM F2996-13	Standard Practice for Finite Element Analysis (FEA) of Non-Modular Metallic Orthopaedic Hip Femoral Stems

**Fig. 4:**
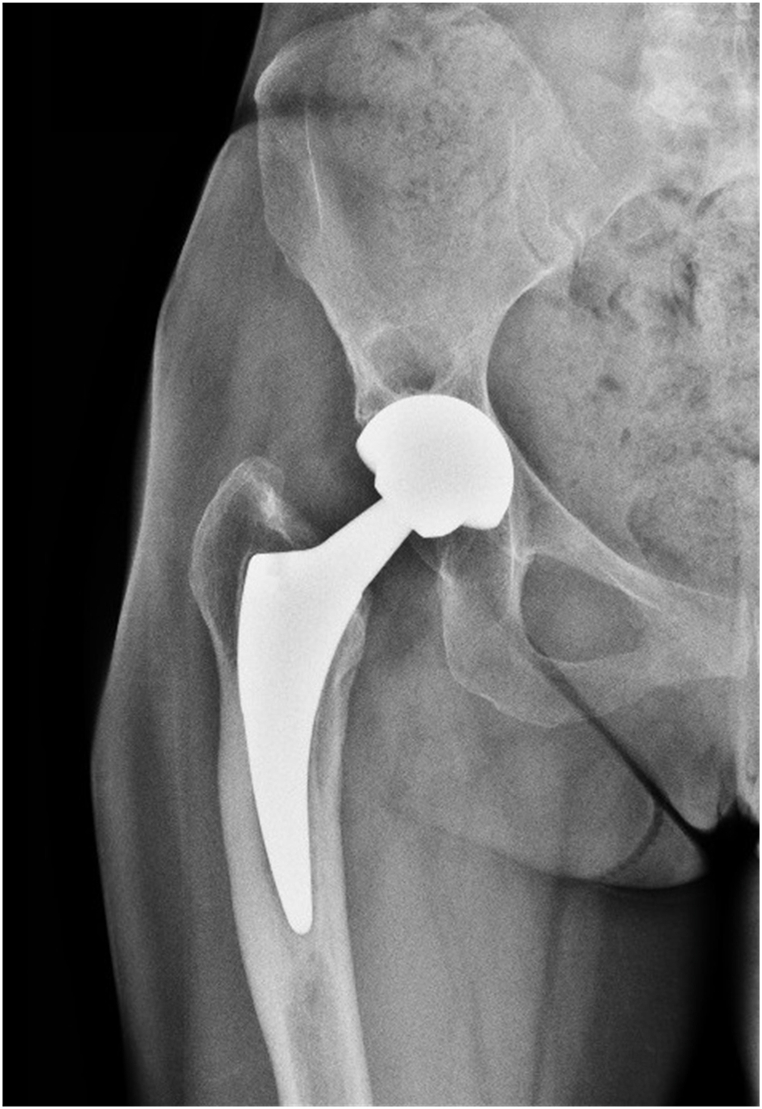
Proximal stress shielding in the Gruen zone 1 and distal cortical hypertrophy in the Gruen zones 2, 3, 4 (Case courtesy of Domenico Nicoletti, Radiopaedia.org, rID: 87,298) [66].

According to the internationally recognized standard ISO 5832, metallic implants for orthopaedic applications can be fabricated using various materials, including stainless steel, Co-Cr alloys, and titanium alloys. Amongst these options, Ti-6Al-4V is the most widely utilised due to its exceptional biocompatibility, ductility, superior corrosion resistance, and lower modulus of elasticity compared to steels [5]. However, even though titanium alloys possess a relatively lower modulus of elasticity, they still exhibit significantly higher values (five to ten times) than bone tissues. Consequently, when a solid implant is present, the transfer of forces to the surrounding bone is reduced. Following Wolf's Law, bone tissue responds to mechanical loads by adapting and remodelling itself. This dynamic characteristic allows bones to strengthen in regions experiencing higher stress and weaken in areas subjected to lesser stress, optimising their structure based on prevailing demands [63,64]. The mechanical property disparity between bone tissue and metallic implants can have significant implications. Firstly, the applied load in the proximal femur may be diminished, leading to bone resorption at the prosthesis-bone interface due to stress shielding. Additionally, increased bone strain at the femoral stem tip may induce distal femoral cortical hypertrophy (DFCH) or the fusiform enlargement of cortical bone in the femoral stem tip (Fig. 4). These complications, including stress shielding and cortical bone enlargement, are widely observed in hip joint implants and substantially heighten the likelihood of implant failure, which can manifest as implant loosening or bone fracture [28,[65], [66], [67], [68]].

**Fig. 5:**
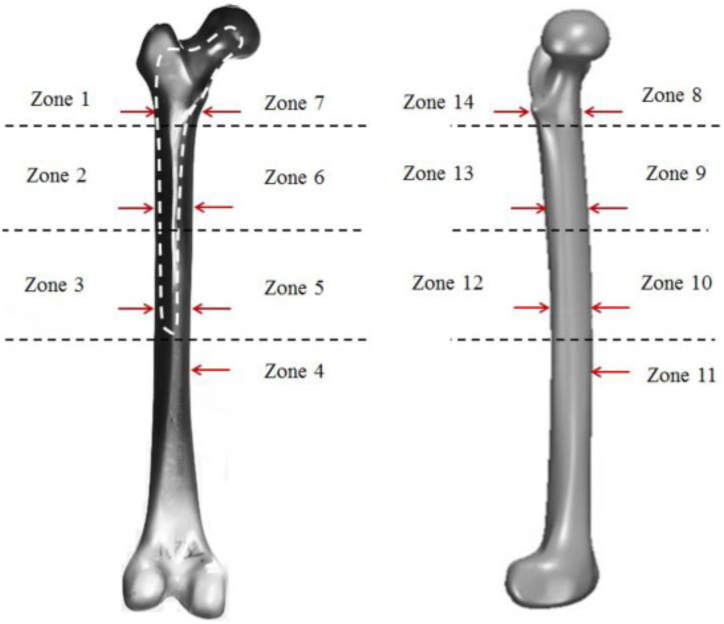
Schematic Gruen zones of the left femur [70].

Fig. 4 shows the femur after implementation, divided into the Gruen zones. Gruen et al. [69] introduced these zones to provide a systematic division of the implanted femur into 14 reference zones (see Fig. 5).

which In the context of hip replacement surgery, the Gruen zones define specific regions that serve as reference points for assessing the stability and fixation of the hip prosthesis with the surrounding bone. These zones, comprising 14 distinct areas along the femoral stem, are instrumental in identifying potential complications, including loosening, stress shielding, or cortical hypertrophy. By employing the Gruen zones, healthcare professionals can effectively communicate and describe any irregularities or complications observed at the prosthesis-bone interface in a standardised manner [65,70,71].

## 4 Porous structures and porous hip joint implants

A viable solution to address the challenges associated with dense metallic implants involves utilising lattice structures as an alternative to solid implants. Advanced additive manufacturing techniques have emerged as valuable tools for fabricating porous structures with exceptional precision, closely matching the intended morphologies. Consequently, numerous studies have explored the diverse characteristics of these lattice structures. Notably, the biocompatibility of lattice structures is significantly enhanced, as they facilitate cell seeding, bone ingrowth, vascularization, permeability, and improved osseointegration [5,20,22,72]. A range of morphologies can be regarded as studying porous structures. Still, the most prevalent and well-known ones are strut-based structures, including the simple cubic (SC), body-centered cubic (BCC), and face-centered cubic (FCC), as well as triply periodic minimal surfaces such as Gyroid, Diamond, and Primitive, or stochastic structures. Table 6 summarises the studies that have examined each of these structures in their respective research endeavours.

**Table 6:** Recent studies on porous structures.

**References**	**Porous structure**	
Ö. Poyraz et al. (2022) [73] P. Müller et al. (2022) [23] L. Bai et al. (2019, 2020) [74] C. Peng et al. (2020) [19] T.A. Alwattar et al. (2020) [75,76] M. Dallago et al. (2019 a, b) [77,78] S. E. Alkhatib et al. (2019) [49] Y. Le et al. (2019) [2] Z.S. Bagheri et al. (2017) [79]	Strut-based (SC, BCC, FCC)	Ö. Poyraz et al. (2022) [73]
Z. Liu et al. (2023) [80] P. Bean et al. (2023) [81] G. Feng et al. (2023) [82] S.A. Naghavi et al. (2022) [7,72] K. Yeranee et al. (2022) [83] E. A. Ramirez et al. (2021) [84] N. Kladovasilakis et al. (2021) [85] J. Corona-Castuera et al. (2021) [58] N. Kladovasilakis et al. (2020) [86] Y. Jin et al. (2020) [87] L. Yang et al. (2019) [88,89] D. Mahmoud et al. (2020) [90] E. Yang et al. (2019) [46]	TPMS (Gyroid, Diamond, Primitive)	a) Iso-surface b) Solid-Network c) Sheet-Network (K. Yeranee et al. (2022) [83])
J. Li et al. (2023) [91] T. Wu et al. (2023) [92] S. Kechagias et al. (2022) [93] N. Tan et al. (2021) [94] L. Tian et al. (2020) [95] Y. Ibrahim et al. (2020) [96] C. Simoneau et al. (2016) [97]	Stochastic structures	S. Kechagias et al. (2022) [93]

Characterising porous structures involves crucial parameters such as porosity, relative density, pore size, unit cell size, and strut thickness. These parameters play a pivotal role in defining the specific attributes of a porous structure. In contrast, changes in these parameters directly influence the mechanical and physical properties, including elasticity modulus, yield strength, plateau stress, energy absorption, and permeability. In biomedical devices, these modifications also impact biological factors, such as cell ingrowth, seeding, vascularization, and osseointegration. To evaluate the compressive properties of porous and cellular metals with a 50 % or more porosity, the compression test outlined in ISO 13314, a widely accepted standard, is employed. This standard is a benchmark for experimental studies, and provides guidelines for defining load and boundary conditions in numerical analysis for estimating elasticity modulus. Recent studies on porous structures and their outcomes, summarised in Table 7, have largely adhered to this standard.

**Table 7:** Recent studies on porous structures.

**Author**	**Aim & Methodology**	**Result**
M. Rezapourian et al. (2023) [98]	Mechanical properties and surface area ratio of Split-P TPMS (FEM & Experiment)	Ti6Al4V Split-P lattices possess the most significant surface area and surface area-to-volume ratio compared to other TPMS structures. This characteristic enables them to exhibit mechanical properties closely resembling trabecular and cortical bones, rendering them appropriate for load-bearing bone implants.
Z. Liu et al. (2023) [80]	Fatigue properties of TPMS structures (FEM & Experiment)	A proposed approach for enhancing the fatigue properties of AM produced Ti6Al4V porous scaffolds, involves combining hot isostatic pressing (HIP) and electropolishing (ELP) as post-treatment methods.
G. Feng et al. (2023) [82]	Mechanical properties and deformation behavior of gradient TPMS structures (FEM & Experiment)	TPMS structures exhibit strain rate sensitivity and outperform traditional porous structures in terms of specific energy absorption. The Diamond structure surpassed the Gyroid structure in terms of elasticity modulus and strength.
P. Bean et al. (2022) [81]	Investigated effective elastic properties and anisotropy index of Gyroid (FEM & Experiment)	The findings demonstrate that despite the cubic symmetry of the gyroid infill, it displays a near-isotropic nature with a low anisotropy index. When the nominal density is set at 50 %, the deviation in the longitudinal modulus is merely 4 % of the modulus of the neat material.
S.A. Naghavi et al. (2022a) [7]	Investigated morphological deviation between as-built and as-designed additively manufactured TPMSs (FEM & Experiment)	The morphological disparity in strut thicknesses and pore sizes within AM-manufactured porous scaffolds displayed variation within the structure, with a standard deviation of approximately 20 μm, except for the horizontal struts in gyroids, where the standard deviation was about 36 μm. By subjecting the scaffolds to postprocessing, via sandblasting, the surface roughness of the initially constructed scaffolds was reduced from 16.98 ± 0.51 μm to 1.21 ± 0.05 μm, which is considered suitable for osseointegration and ensures long-term bone-implant fixation. The elasticity modulus of the TPMS investigated ranged from approximately 6.81 to 7.59 GPa.
S.A. Naghavi et al. (2022b) [72]	Investigated compressive, tensile, bending, and torsional stiffness and strength of the TPMS scaffolds (FEM & Experiment)	TPMSs within the 54–72 % porosity range exhibit physical and mechanical properties that closely resemble those of cortical bone. This similarity makes them well-suited for implementation in orthopaedic treatments.
L. Bai et al. (2021) [74]	Compressive and fatigue behavior of graded strut-based structures (FEM & Experiment)	The findings indicate that the vertically graded (VG) structure demonstrates exceptional mechanical properties and energy absorption capacity. Compared to the uniform structure, the VG structure exhibits a significant increase of 17.53 % in elastic modulus and a notable enhancement of 59.43 % in energy absorption. On the other hand, the radial-graded (RG) structure shows a compression response similar to that of the uniform structure.
E. A. Ramirez et al. (2021) [84]	An analytical investigation to explore how the design parameters of TPMSs affect the relative density	The findings reveal a linear correlation between the relative density of the pattern and its thickness, whilst the relationship with length is non-linear. As a result, the study proposes relative density equations that can be implemented for topological optimisation procedures.
N. Kladovasilakis et al. (2021) [85]	Investigated the mechanical behavior of TPMS structures (FEM & Experiment)	The Schwarz Primitive structure was affected by the size effect, leading to decreased structural integrity and diagonal failure due to bending and shear stresses. However, it exhibited the highest energy absorption capacity among the TPMS structures, withstanding substantial loads even after yielding. In contrast, the gyroid structure demonstrated superior stiffness, elastic modulus, and remarkable rigidity compared to other TPMS structures.
D. Mahmoud et al. (2021) [90]	Fatigue properties of uniform and graded porosity gyroids (FEM & Analytical study & Experiment)	Radially graded gyroids exhibited higher compressive strength than uniformly graded structures, making them more advantageous for load-bearing implants. Additionally, thin strut designs displayed superior normalised fatigue properties compared to thick and graded strut parts. The higher fatigue strength in thin structures can be attributed to their increased ductility, reduced internal defects, and smaller surface area per strut. These factors collectively contribute to delayed crack initiation and slower crack propagation compared to other designs.
Y. Jin et al. (2020) [87]	Mechanical performance of multi-sheet TPMS with various functional gradients (FEM & Experiment)	The proposed TPMS-based branched structures hold significant potential for offering versatile properties, including tunable permeability and mechanical characteristics.
C. Peng et al. (2020) [19]	Mechanical and fatigue properties of strut-based structures (FEM & Experiment)	The number of unit cells within a lattice structure has a minimal impact on elastic properties, but it significantly influences plastic behaviors. The normalised fatigue behavior of SC and SC-BCC remains unaffected by relative density, whilst FCC and BCC structures are sensitive to changes in relative density. However, the research findings indicate that increasing relative density enhances fatigue resistance for all lattice structures investigated.
T.A. Alwattar et al. (2020) [75]	Developed a quasi-elastic equivalent model for an inside BCC structure (FEM & Experiment)	The comparison between experimental data and finite element simulation results of the entire lattice and equivalent solid models reveals a strong agreement. Particularly within the linear elastic limit, the stress-strain behavior obtained from the finite element analysis (FEA) models closely matches the experimental observations.
L. Yang et al. (2019) [88]	Mechanical properties of uniform and graded gyroid solid network (FEM & Experiment)	The compression experiments reveal that cellular structures with a density gradient oriented perpendicular to the loading direction, exhibit deformation behaviours comparable to those observed in configurations with uniform density. Additionally, a density gradient perpendicular to the loading direction improves the Young's modulus, plateau stress, and energy absorption capacity of the cellular structure.

**Fig. 6:**
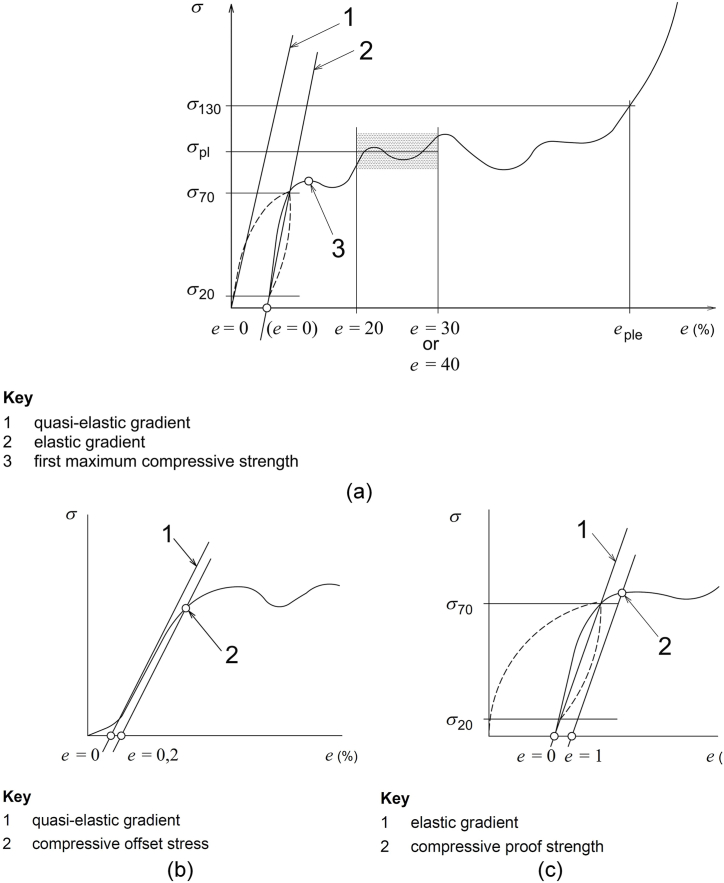
(a) ISO13314 method for determining the characteristic value from compression test using stress-strain curve, (b) option 1: determination of quasi-elastic gradient and compressive offset stress, (c) option 2: determination of elastic gradient and compressive proof strength.

ISO 13314 outlines the requirements for test specimens, which can take either rectangular or cylindrical shapes. This standard defines the various characteristic values extracted from the stress-strain curve (see Fig. 6(a)), including plateau stress, energy absorption efficiency, and plateau end stress and strain. Additionally, it provides two options for calculating quasi-elastic gradient and compressive offset stress, or alternatively, elastic gradient and compressive proof strength, as depicted in Fig. 6(b) and (c), respectively.

Two significant parameters that correspond to a porous structure's elasticity modulus (stiffness) and yield strength are the quasi-elastic gradient and the compressive offset stress. Researchers often use the Gibson-Ashby model to establish a comprehensive relationship between the mechanical properties and porosities of porous structures [18]. This model is a fundamental framework for understanding the interplay between porosity and mechanical behaviour. According to the Gibson-Ashby model, a porous structure's relative elasticity modulus and relative yield strength can be expressed as power functions of the relative density. Equations (8), (9)) present the mathematical representations of this relationship:

(8) $$E^{*}=C_{1}{\left(\rho^{*}\right)}^{n}$$

(9) $$\sigma^{*}=C_{2}{\left(\rho^{*}\right)}^{m}$$

Herein, E∗, σ∗, and ρ∗ denote the relative elasticity modulus, relative yield strength, and relative density, respectively, and C_1_, C_2_, n, and m are specific factors that depend upon the particular geometry under consideration. These coefficients can be determined through experimental investigations or derived from numerical analysis.

Using the Gibson-Ashby model provides researchers with valuable insights into the influence of porosity on the mechanical properties and behavior of porous structures. Additionally, it allows for the distinction between bending-dominated and stretching-dominated structures. Specifically, the structure tends to exhibit stretching-dominated behavior as the power value approaches one. In contrast, a value exceeding two indicates bending-dominated behavior. This understanding of the mechanical response aids in designing, optimising, and customising porous materials across various applications, including structural engineering, biomaterials, and energy storage. To summarise the distinctive behaviours of these two structural types, Table 8 provides an overview of their characteristics [99].

**Table 8:** Porous mechanical behavior characteristics.

**Characteristic**s	**Bending-Dominated Behaviour**	**Stretching-Dominated Behaviour**
Gibson-Ashby Power (n)	n ≥ 2	n ≈ 1
Connectivity	Low connectivity with struts and surfaces	High connectivity with struts or surface
Deformation Mechanism	Struts/surfaces bend upon application of loads	Struts/surfaces elastically stretch under applied loads
Elastic Modulus	Exponential relation between effective elastic modulus and relative density	A linear relationship between elastic modulus and relative density
Stiffness	Low stiffness	Higher stiffness compared to bending-dominated structures
Peak Strength	Low peak strength due to bending of struts/surfaces	Higher peak strength due to connectivity
Structure Connectivity	Low connectivity of the structure's elements	High connectivity in struts or surface
Stress-Strain Curve	Constant stresses create a plateau section in the stress-strain curve	Decrease in strength after peak strength
Plastic Deformation	Deforms plastically until the densification section	Plastic buckling or brittle fracture
Energy Absorption	Remarkable energy absorption from the plastic section	Moderate energy absorption capabilities
Applications	Suitable for impact applications	Excellent for applications requiring enhanced structural integrity

When considering implementing porous structures for biomedical applications, crucial parameters, including unit cell size, porosity, and pore size, assume greater significance due to their direct impact on vital aspects such as cell seeding, vascularization, and permeability. These factors play a pivotal role in expediting the processes of osseointegration and implant stability. However, determining the precise pore size and porosity values remains a topic of ongoing debate. Nevertheless, several studies have suggested ranges that hold promise for various biomedical applications. Table 9 presents the proposed pore size and porosity ranges that have shown potential suitability for biomedical applications.

**Table 9:** Recommended pore size and porosity.

**Author**	**Pore size**	**Porosity**	**Benefit**
R. Alkentar et al. (2023) [100]	500–750 μm	50–90 %	Review paper: a conclusion by synthesising the existing literature
J. Triyono et al. (2020) [101]	Above 300 μm	–	Facilitate optimal vascularization and enhance the attachment of bone cells.
Q. Wang et al. (2020) [102]	100–1000 μm	30–80 %	Enhance the initial osseointegration period.
D. Barba et al. (2019) [103]	300–600 μm	Above 50 %	Facilitate bone ingrowth and simultaneously enhance osseointegration, vascularization, and cell growth.
S. Barui et al. (2017) [104]	Min 100 μm Larger than 400 μm	–	It is crucial to incorporate interconnected pores exceeding 400 μm in size to facilitate vascularization. In contrast, a minimum pore size of 100 μm is necessary for promoting osteogenesis.
K. Arabnejad et al. (2012) [1]	50–800 μm	Above 40 %	Facilitate and promote bone ingrowth.
S. Van Bael et al. (2012) [105]	500 μm	–	Enhance the initial cell seeding, colonization, and attachment processes.
A. Fukuda et al. (2011) [106]	500 μm	–	It is highly recommended for porous biomedical devices.
S. Wu et al. (2013) [107]	700 μm	–	Promote and enhance osseointegration.
F. P. Melchels (2010) [21]	300–800 μm	–	Capillary formation necessitates a minimum pore size of 300 μm. Additionally, no statistically significant difference in bone ingrowth and bone formation has been observed in scaffolds with up to 800 μm pores.
P Heinl et al. (2008) [15]	Above 100 μm	–	A minimum pore size is essential to facilitate cell penetration, tissue ingrowth, vascularization, and effective nutrient delivery to the core of regenerating tissue.
S. Harrysson et al. (2008) [108]	50–800 μm	–	This feature is highly suitable for promoting bone ingrowth and is often considered crucial when designing porous structures for femoral stem applications.

The numerous advantages of porous structures have prompted researchers to increasingly employ lattice morphologies in the development of biomedical devices, with a specific focus on hip joint implants. These implants are particularly susceptible to complications arising from higher loads, such as stress shielding and cortical hypertrophy. The Gibson-Ashby model has provided valuable insights into the relationship between porosity, relative density, and mechanical properties, indicating that an increase in porosity or decrease in relative density can effectively reduce the relative elasticity modulus or stiffness of the implant. As a result, the elasticity modulus of porous structures can be precisely tailored to align with that of the host bone. In light of this, extensive investigations have explored the feasibility of adjusting the elasticity modulus to match the mechanical properties of the local bone. This has been achieved by implementing gradient or uniform lattice morphologies in the design of hip joint implants. The primary objective of these studies is to assess the potential of these lattice-based designs in mitigating the complications mentioned associated with hip joint implants, such as stress shielding and cortical hypertrophy. By fine-tuning the porosity, researchers aim to optimise the implant's mechanical properties, ultimately promoting improved osseointegration, reducing stress concentrations, and enhancing long-term implant performance.

**Fig. 7:**
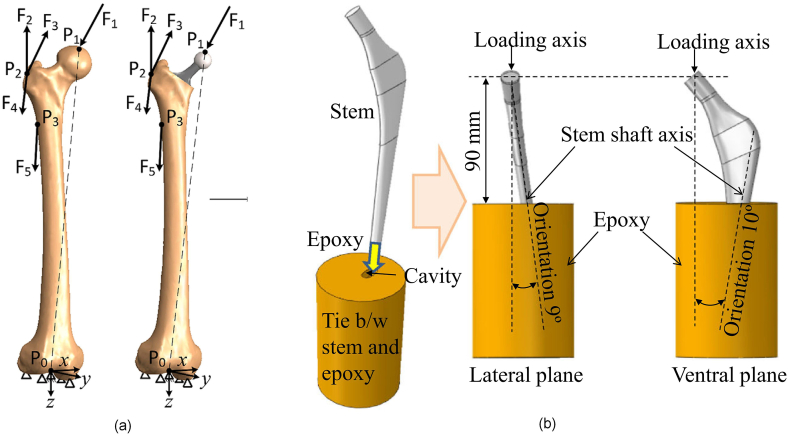
Load and boundary condition (a) Option 1: In-Vivo [57] (b) Option 2: According to ISO 7206-4 [116].

Additionally, the advent of state-of-the-art manufacturing technologies, such as diverse powder bed fusion metallic additive manufacturing methods, including Selective Laser Melting (SLM), Selective Laser Sintering (SLS), and Electron Beam Melting (EBM) [109], has revolutionized the control over microarchitecture within porous structures. These advanced techniques enable the production of struts and pores with minimum dimensions of around 100 μm, meeting the stringent requirements for biomedical devices. As a result, there has been a significant upsurge in research efforts to replace solid metallic devices with porous counterparts. Various methodologies have been explored to achieve this objective, ranging from integrating uniform porosity in the distal part of the stem, to incorporating uni- or multi-directional gradient porosity and leveraging topology optimisation-based approaches. To assess the stress distribution on the stem under diverse loading conditions, numerical analysis utilising the Finite Element Method (FEM) has become the predominant technique in these studies. The applied loads and boundary conditions are typically simulated by either modelling the hip joint stem insertion into a femur model [14,23,44,45,[49], [50], [51],^53^,^56^,^57^,[110], [111], [112], [113]], or by following well-established standards, such as ASTM F2996 (Standard Practice for Finite Element Analysis (FEA) of Non-Modular Metallic Orthopaedic Hip Femoral Stems) and ISO 7206-4 (Determination of endurance properties and performance of stemmed femoral components) [51,53,55,94,110,[114], [115], [116], [117], [118]], which are specifically designed for hip joint non-modular implants. Fig. 7 visually illustrates these two approaches as Option 1 and Option 2, respectively.

By selecting option 1, it becomes possible to accurately capture the influence of the stem on stress distribution within the bones. This approach allows for evaluating critical factors, such as the stress-shielding phenomenon and micromotion between the stem and the bones. However, it should be noted that this option introduces complexity to the model, as it necessitates using a verified femur model and accurate implant placement. Additionally, the simulation time is extended due to the increased intricacy of the model. Option 2 offers the advantage of reduced simulation time compared to option 1. However, the stress distribution on the components may deviate from the in-vivo conditions, particularly when the design is developed based on strain energy or topology optimisation.

Nevertheless, option 2 can serve as a valuable tool for verifying the feasibility of the developed design and assessing its ability to meet the minimum requirements stipulated by relevant standards. Ultimately, the choice between these two options depends on the study's particular objectives, and the trade-offs between accuracy, complexity, and computational efficiency. Careful consideration should be given to selecting the most appropriate approach based on the research goals and limitations.

To provide a comprehensive overview of the progress made in this field, Table 10 summarises the key findings from previous studies investigating the implementation of porous structures in hip joint implant design.

**Table 10:** Recent studies implementing porous structures for hip joint implant.

**Author**	**Porous type**	**Result**
P. Muller et al. (2024) [119]	Stem with topology optimised strut-based lattice	Implementing a porous implant instead of a solid stem significantly increased the stress transferred to the femur, resulting in a reduction in the stress deviation between the healthy bone and the implanted bone.
J. Moghariya et al. (2024) [120]	Uniform Gyroid and Split-P porous hip stem (TPMS)	Both TPMS structures reduced stress shielding; however, the Gyroid structure demonstrated superior stress distribution within the femur, making it a better option for implantation.
P. Bolshakov et al. (2024) [121]	Stem with optimised gradient-density cube unit with spherical hole	Structurally focused, the analysis underscores a 9 % and 11 % volume reduction in implants, with consideration for stress shielding omitted. Maximum von Mises stress increased for one type, whilst remaining unchanged for the initial design. The load-bearing capacity was maintained. Optimised implants minimised material usage, expedited design iterations, and enabled intricate functional structures through additive manufacturing.
Z.F.M. Salaha et al. (2023) [122]	Stem with uniform Voronoi and Gyroid structures (TPMS)	The Voronoi lattice is the top choice for hip implants due to its lightweight design and low stress. The Gyroid and Voronoi implants showed displacement differences of around 32 % and 16 %, respectively, suggesting reduced rigidity that helps alleviate stress shielding.
M. Rana et al. (2023) [59]	stem incorporates four distinct strut based porous structure categories, each comprising combinations of positive and negative Poisson's ratios within its various regions	By utilising a multi-objective optimisation strategy in stem design, the stress shielding across the four Gruen zones of the implanted femur bone was reduced by 38 %.
N. Rahmat et al. (2023) [123]	Stem with axially graded IWP and Gyroid structures (TPMS)	The stem with an IWP structure exhibits higher stress shielding compared to the Gyroid at the same volume fraction. The decreasing axially graded Gyroid demonstrates superior stress transfer, particularly in the proximal medial section. This configuration led to a reduction in stress shielding by 14.42 % in Gruen zone 6 and 13.37 % in Gruen zone 7 for this model.
S.A. Naghavi et al. (2023) [110]	Uniform TPMS porous hip stem	The stress shielding effect in the proximal femur (Gruen zones 1 and 7) was significantly reduced when using the porous stem, with only 37 % compared to 68 % observed in the solid stem model. Moreover, the porous stem demonstrated a substantial reduction of approximately 70 % in total SSI (Stress Shielding Index), across all Gruen zones 1–7, compared to the solid stem model.
Cortis et al. (2022) [45]	Uniform porous BCC hip stem	The study results demonstrated an 11 % reduction in stress shielding in Gruen zone 6 and a 25 % reduction in Gruen zone 7.
N. F. Al Zoubi et al. (2022) [53]	Porous SC hip stem	The stems exhibiting an average porosity of 70 % were identified as the most suitable match for the mechanical properties of intact bone. Conversely, the stems with 30 % and 50 % average porosity displayed moderate interfacial micromotions, allowing for sufficient stress transfer to the bone without experiencing yield.
X. Gao et al. (2022) [44]	Kriging algorithm-based porous stem	The parameterised design method proposed in this study for the porous implant with a gradient elastic modulus, resulted in an average increase of 17.1 % in strain values on the femoral surface.
E. Garner et al. (2022) [124]	Stem with uniform and optimised graded structures with assembly of negative, neutral, and positive Poisson's ratios	The optimised design significantly reduces bone resorption compared to the uniform porous implant, and minimised interface stress relative to the solid implant. Additionally, it promoted much lower bone remodelling compared to the solid implant.
Y.K. Cheah et al. (2022) [125]	Stem with uniform strut based, FBCCz and Octet-truss lattice structures with different porosities	The proposed octet-truss lattice, featuring a 25 % porosity in a specific region of the stem (with a moderate volume fraction of the stem), emerged as the most suitable design. This design enhances the open surface area by over two times compared to the solid implant, facilitating superior osseointegration.
K. M. Abate et al. (2021) [115]	Porous Vintiles lattice topology	The optimised cellular hip implant exhibited a significantly lower stiffness, measuring 62 % less than its solid counterpart. Additionally, the cellular implant demonstrated a remarkable weight reduction, 50 % lighter than the solid implant. These findings highlight the potential benefits of using cellular implants, particularly those with 56 % and 58 % porosities, in orthopaedic and prosthetic applications, as they can enhance osseointegration.
N. Tan et al. (2021) [94]	A topology-optimised stem by incorporating a stochastic porous structure and a selectively hollowed approach	The measured stiffness values for the two optimised stems, namely the porous and selectively hollowed implants, were nearly identical. Both designs exhibited significant reductions in equivalent stiffness, with the porous implant showing a 39 % reduction, and the selectively hollowed implant showing a 40 % reduction, compared to the solid implant.
N. Kladovasilakis et al. (2020) [86]	Stems based on topology optimisation of TPMS structures	The optimal geometry of the hip implant was achieved by incorporating functionally graded lattice structures, which improved stress behaviour. This design allowed the hip implant to withstand up to twice the in vivo loads, indicating its enhanced mechanical strength and durability.
S. Wang et al. (2020) [111]	Porous diamond cubic hip stems feature a distally increased porosity axial gradient and an inward increased porosity radiant gradient	The utilisation of porous stems with 50 % and 60 % porosity models was found to elevate the risk of excessive micromotions, potentially resulting in implant instability.
N. Kladovasilakis et al. (2020) [126]	Hip implants utilize solid materials and lattice structures in their composition.	The results demonstrated that topology-optimised hip implants incorporating lattice structures were mechanically functional and safe to use, with the ability to withstand loads associated with various movements.
H. Mehboob et al. (2020a) [55]	Two uniform BCC hip stems with different stiffness values: 31.5 GPa and 53.8 GPa.	The proposed porous titanium (Ti) stems, featuring an inner porous BCC structure and an outer dense shell, significantly reduced stress shielding compared to solid stems. Additionally, these porous Ti stems showed promising bone formation. Lower-stiffness stems exhibited less stress shielding, higher initial micromotions, and improved tissue differentiation compared to higher-stiffness stems.
H. Mehboob et al. (2020b) [116]	A porous hip stem with a BCC structure. The hip stem was evaluated across five different thicknesses of outer dense shells and six porosity levels.	Fully porous stems without dense shells are prone to failure under fatigue load. Therefore, it is recommended to use porous stems with a shell thickness of 1.5 mm and 2 mm for all porosity levels ranging from 18 % to 90 %. A 1 mm shell thickness is suitable for 18 % and 30 % porosities, while a 0.5 mm shell thickness is sufficient for 18 % porosity. By employing these recommended shell thicknesses, a notable reduction in stress shielding of approximately 28 % was achieved.
S. E. Alkhatib et al. (2019a) [49]	Body-centered cubic (BCC) with different strut sizes	The porous stems with a thickness of 0.87 mm strut demonstrated the lowest stress shielding signals, measuring less than 5 %, across all loading conditions.
S. E. Alkhatib et al. (2019b) [50]	Homogeneous porous (HGP), Functionally graded porous (FGP), Body‐centered cubic (BCC) structures stems	Functionally Graded Porous (FGP) stems transferred significantly higher stresses to the femur, specifically in Gruen zone 7, ranging from 120 % to 170 % higher than solid stems. In contrast, Homogeneous Porous (HGP) and FGP stems exhibited lower stresses, ranging from 12 % to 34 % lower than the dense stem. Regarding micromotions, stems with an overall porosity of 80 % displayed the highest values, ranging from 105 μm to 147 μm. Conversely, stems with an overall porosity of 20 % exhibited the lowest micromotions, ranging from 42 μm to 46 μm.
Y. Wang et al. (2018) [14]	A Porous tetrahedron hip stem with topology optimised distribution.	The numerical results demonstrated that the optimised porous implant had significantly lower bone loss than the fully solid implant (42 % less) and the porous implant with uniform density (79 % less). These findings highlight the potential of the optimised porous implant to minimize bone loss and improve long-term stability and integration.
C. Sun et al. (2018) [56]	Gradient modulus distribution	The optimised femoral stem showed a high global safety factor of 11.3, indicating its strength and safety. Around 26.4 % of the stem elements were designed as solid, contributing to its structural integrity. Additionally, the optimised design resulted in a significant reduction of 40 % in bone volume with density loss compared to a solid stem. This reduction suggests that the optimised design may help minimize bone density loss and improve the implant's long-term stability.
B. Jetté et al. (2018) [114]	Diamond lattice structure	A porous stem exhibits a considerable reduction in stiffness of approximately 30 % compared to its solid counterpart. Furthermore, diamond lattice structures perform better than stochastic cell structures with the same porosity level.
He et al. (2018) [51]	Porous Face and Body-Centered Cubic with Vertical Struts Unit Cell	The optimised design achieved a significant reduction of over 50 % in stress shielding compared to a conventional generic implant. Additionally, the fatigue life of the optimised design met the ISO Standards, ensuring its durability and longevity.
H. Mehboob et al. (2018) [117]	Hip stems with three different lattice structures (SC, diamond, and BCC) and variations in unit cell sizes and numbers	The porous stem composed of the BCC cellular microstructure, accounting for approximately 50 % of the total stem volume, provided the best mechanical performance and alignment with the bone's mechanical behaviour.
H. M. A. Kolken et al. (2017) [127]	Hybrid meta-biomaterials hip implants (meta-implants)	The novel meta-implant designs were observed to generate compressive forces along the contact lines between the implant and the adjacent bone. Based on Hoffman's criterion, this compression phenomenon effectively decreased the likelihood of bone-implant interface failure. Additionally, it acted as a barrier, preventing the infiltration of wear particles into the interface space, thereby reducing stress-shielding effects and promoting enhanced bone ingrowth.
S. Arabnejad et al. (2016) [57]	Porous tetrahedron hip stem	An implant that is fully porous and features an optimised material micro-structure has the potential to reduce the amount of bone loss caused by stress shielding by a remarkable 75 %, compared to a fully solid implant.
O. L. A. Harrysson (2008) [108]	Hip stems with non-stochastic mesh structures	Based on the findings from numerical analysis, the hip stem with a lower bend modulus achieved a more homogeneous stress distribution in the proximal region of the femur.

**Fig. 8:**
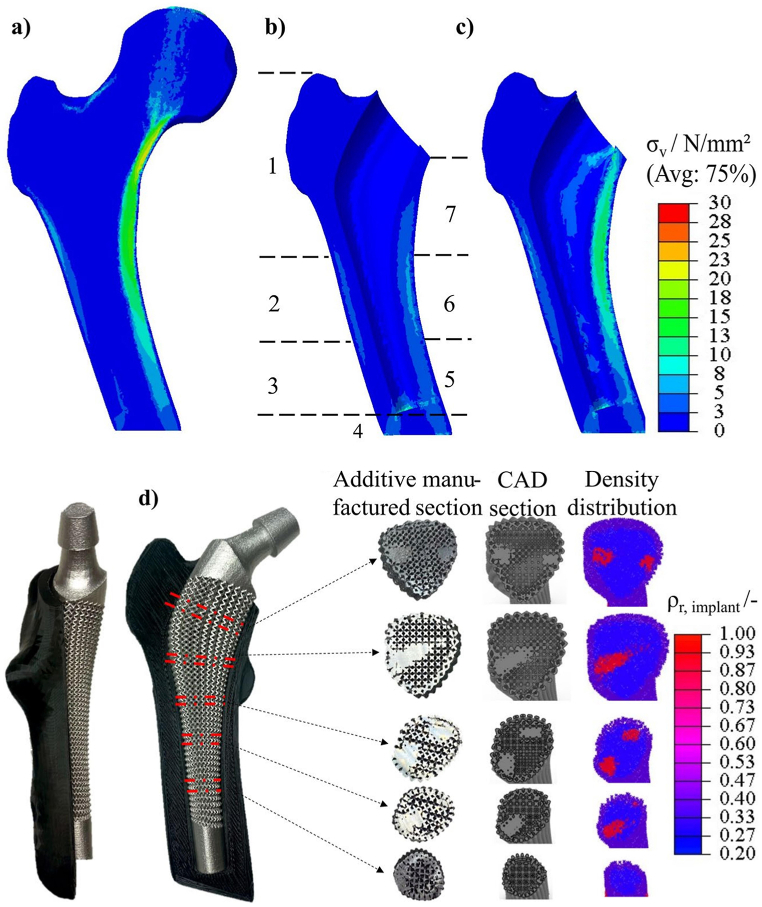
(a) Stress distribution in healthy bone; (b) Bone with solid implant; (c) Bone with topology optimised porous implant; (d) Additively manufactured topology optimised porous implant [119].

To visualise the effectiveness of porous structures in the design of hip implants compared to solid implants, the results of several case studies are presented. Fig. 8 illustrates the stress distribution on the femur in three different scenarios: a healthy bone, a femur with a solid implant, and a femur with a density-based, topology-optimised lattice implant, as studied by P. Muller et al. (2024) [119]. The findings indicate that the topology-optimised lattice implant significantly enhances stress distribution within the cortical bone, closely approximating the distribution in a healthy bone. Specifically, stress deviation between a healthy bone and a femur with an optimised porous implant can be reduced by 81 % and 66 % in Gruen zones 6 and 7, respectively, compared to a femur with a solid implant.

**Fig. 9:**
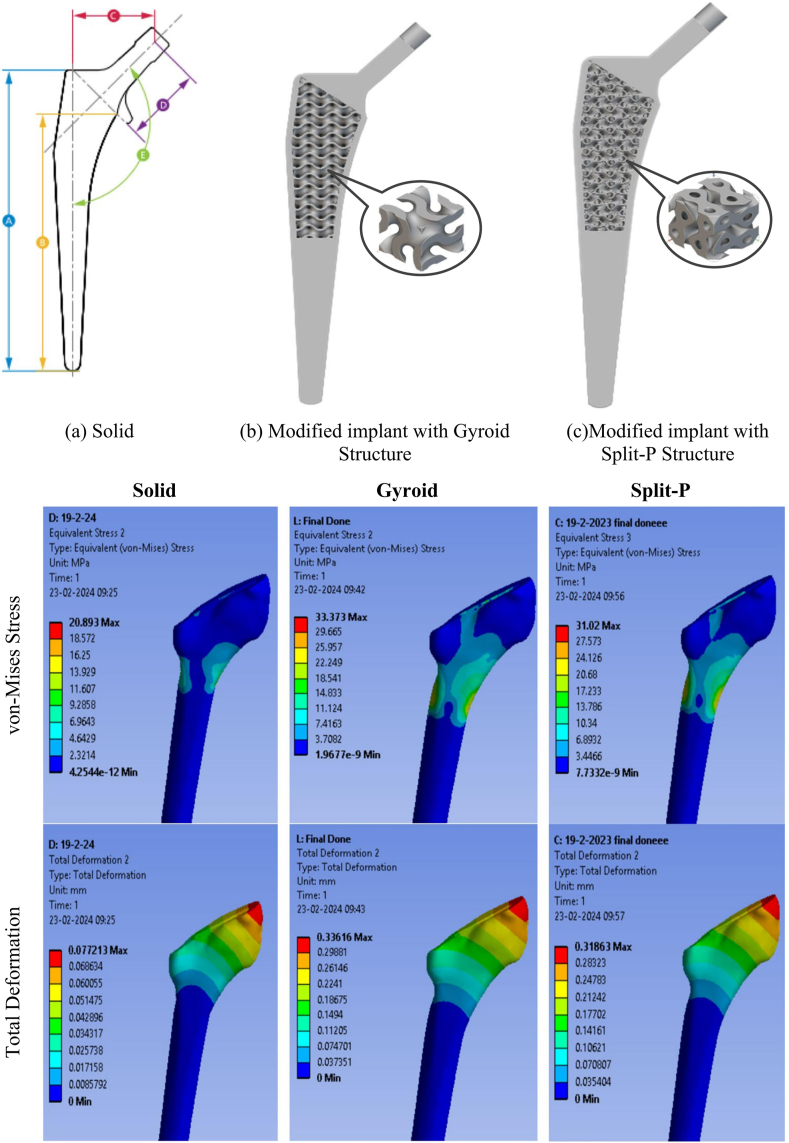
Developed solid and porous implants, and their influence on the stress distribution and deformation of the femur [120].

In a study conducted by J. Moghariya et al. (2024) [120], porous implants utilising TPMS structures with approximately 70 % uniform porosity were developed to mitigate the stress shielding phenomenon. The design incorporated a solid shell surrounding the porous section, which, whilst not enhancing biological parameters such as osseointegration, cell seeding, and vascularization, did effectively reduce stiffness and mitigate stress shielding. As illustrated in Fig. 9, replacing the solid implant with porous implants resulted in increased stress distribution and total deformation of the femur by 51.58 % and 85.39 %, respectively, for implants with gyroid structures, and by 121.29 % and 89.82 %, respectively, for implants with Split-P structures. This led to greater stress transfer to the femur, thereby reducing stress shielding.

**Fig. 10:**
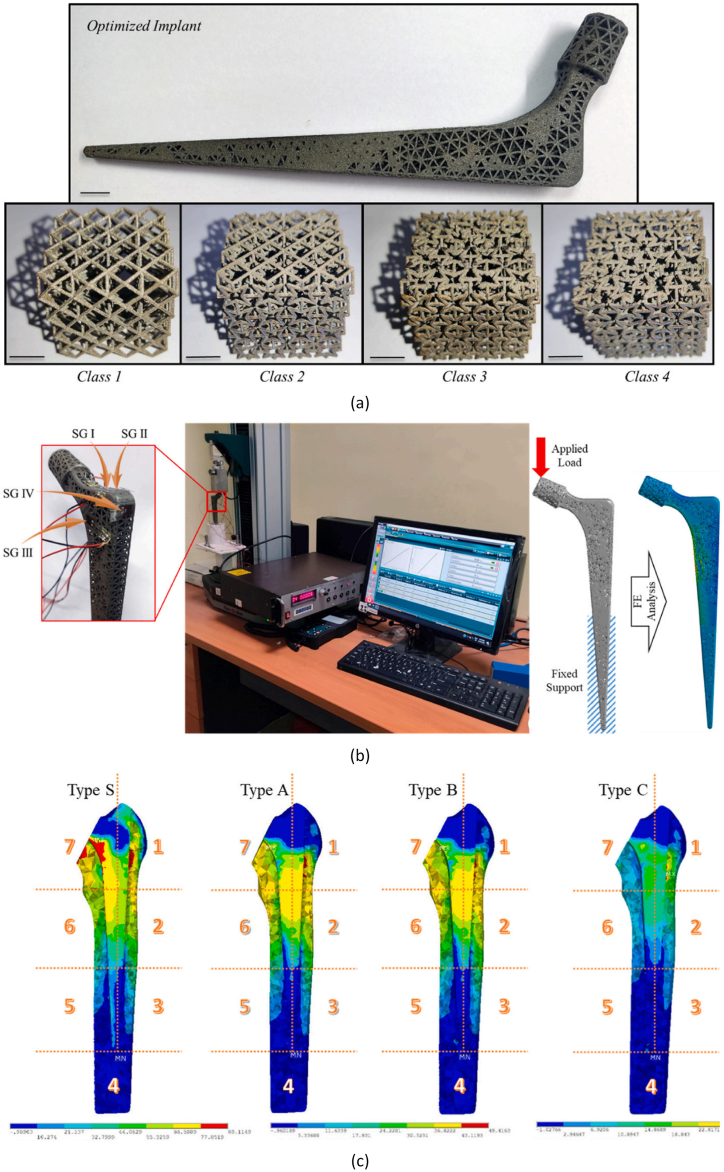
(a) Developed porous implants; (b) Experimental setup; (c) Stress shielding inside femur for four types of implants [59].

M. Rana et al. (2023) [59] developed four different classes of strut-based orthotropic auxetic implants with optimised porosities, which were used to create three types (A, B, C) of implants with various combinations of structures and porosities. These were compared to a solid implant (type S) to address the problem of stress shielding. As depicted in Fig. 10, the developed implants were successfully additively manufactured and experimentally verified. According to their numerical studies, the average stress shielding in Gruen zones 1, 2, 6, and 7 was reduced from 56 % with the solid implant to 18 % with the porous implants.

**Fig. 11:**
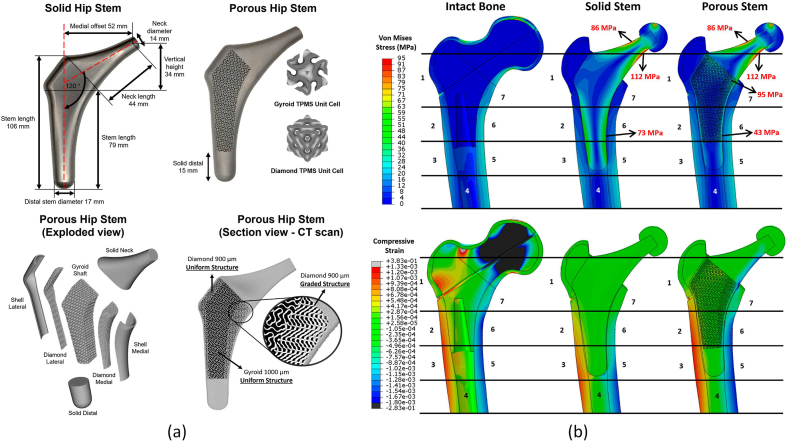
(a) Solid and porous implants; (b) Stress and strain distribution in the femur [110].

A hybrid design of TPMS structures, comprising partly uniform gyroid mixed with diamond unit cells, was developed by S.A. Naghavi et al. (2023) [110] to achieve a low-stiffness porous hip implant. The design was manufactured using additive manufacturing and underwent fatigue testing, with its effect on stress distribution in the femur investigated according to ISO 7206-4 standards. Both loading options were implemented in this study for numerical and experimental approaches. As shown in Fig. 11, the stress shielding index and bone loss in the femur with the porous implant were reduced by 70 % and 61 %, respectively, compared to the solid implant. Fatigue failure occurred after approximately 457,000 cycles, which is not sufficient considering the minimum endurance cycle of 10^6^ cycles. Therefore, further efforts are required to optimise the design to meet the minimum required cycles from a fatigue strength point of view.

The findings from these studies present promising prospects for implementing a porous structure in biomedical devices, particularly in the case of hip joint implants. Manufacturing strut-based and TPMS structures within morphological dimensions suitable for biological applications, such as osseointegration, cell seeding, vascularization, and permeability, presents significant challenges. Strut-based structures often feature sharp edges and corners that are prone to stress concentration, which can be detrimental to fatigue resistance. Additionally, these structures require precise adjustments to be fabricated without supports in additive manufacturing processes. Despite these issues, strut-based designs are relatively straightforward to model and analyse during design and numerical investigation. In contrast, TPMS structures, characterised by their self-supporting geometries, offer smooth surfaces and excellent strength-to-weight ratios, which help mitigate stress concentration and enhance mechanical performance. However, the inherent complexity of TPMS structures leads to increased manufacturing costs and difficulties in modelling and optimisation, as well as extended computation times for numerical analysis. Thus, whilst TPMS structures provide superior mechanical properties and are well-suited for additive manufacturing, they present a trade-off in terms of complexity and cost compared to the simpler strut-based designs.

**Fig. 12:**
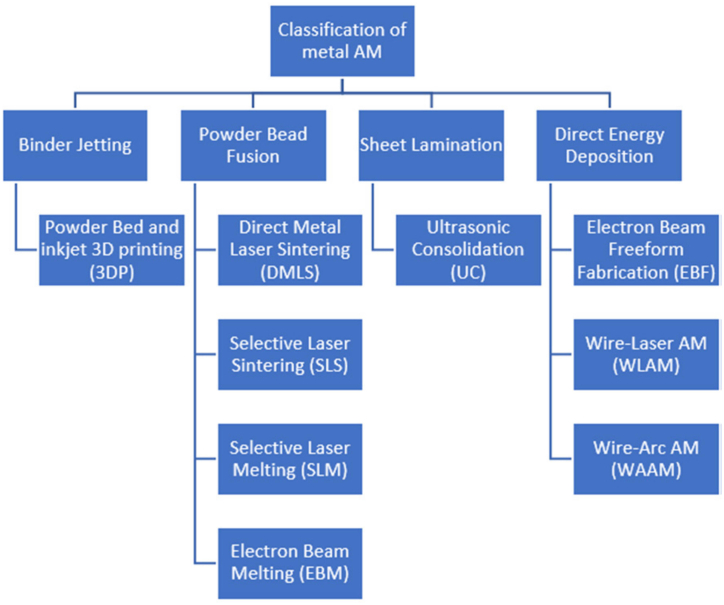
Classification of metal additive manufacturing methods [109].

In light of these challenges, the role of additive manufacturing (AM) becomes pivotal in advancing both strut-based and TPMS structures. Additive manufacturing technologies offer unprecedented flexibility in creating complex geometries and intricate designs that are difficult to achieve with traditional fabrication methods. Recent studies highlight how AM can overcome the limitations of strut-based structures, by enabling the precise production of geometrically complex lattice configurations without the need for additional support structures. Similarly, TPMS structures benefit from AM's ability to fabricate self-supporting geometries with high resolution and accuracy, addressing some of the complexities associated with their design and optimisation. The continuing evolution of AM technology is likely to further enhance the capabilities of both structural approaches, driving innovation in fields such as biomedical implants. Fig. 12 shows a classification of metal additive manufacturing technologies [109].

Table 11 summarises studies which have utilised additive manufacturing for the fabrication of porous structures for biomedical applications.

**Table 11:** Studies utilising AM for fabricating porous structures.

**Author**	**AM**	**Investigation/Procedure**	**Result**
Z. Liu et al. (2023) [80]	EBM (TPMS)	Examined the effects of hot isostatic pressing (HIP) and electropolishing (ELP) on the surface quality, internal microporosity, and microstructure of porous scaffolds fabricated via Electron Beam Melting (EBM)	This study proposes a post-treatment approach, combining HIP and ELP to enhance the fatigue properties of additive manufactured Ti6Al4V porous scaffolds. This method effectively reduces surface roughness, minimises microporosity, and relieves residual stress within the Ti6Al4V porous scaffolds.
M. Rezapourian et al. (2023) [98]	SLM (TPMS)	Investigated the capability of SLM in fabricating Split-P TPMS lattice structures. Scanning Electron Microscopy (SEM) was employed to evaluate the manufacturability and accuracy of the 3D-printed Split-P lattice structures.	For certain porosities and cell morphologies it is crucial to incorporate support structures to stabilise overhanging features and components of the build platform.
S. A. Naghavi et al. (2022) [7]	SLM (TPMS)	Examined the morphological deviations between the designed and printed scaffolds. The samples were subjected to heat treatment and subsequently sandblasted with quartz sand to remove loosely bonded particles.	Morphological deviations were attributed to the over-melting of the struts, leading to horizontal and vertical struts being thicker than their intended dimensions. Horizontal struts exhibited more significant deviations—around 64 % for the gyroid and 34 % for the diamond—compared to vertical struts, which had deviations of approximately 4 % for the gyroid and 2 % for the diamond.
A. EI. Elmi et al. (2020) [128]	SLM (Strut-based structures)	Examined defects in Ti–6Al–4V octet truss lattice materials produced through selective laser melting.	Surface defects predominantly manifested as bonded particles, averaging 40 μm in size, which impacted the surface quality of the fabricated parts. Microstructural defects, including pores, cracks, and poor bonding, were primarily observed in the struts.
M. Dallago et al. (2019) [78]	SLM (Strut based structures)	Examined the discrepancy between the designed and printed strut thicknesses in regular cubic cell lattices.	The vertical struts exhibited the greatest deviation from their intended dimensions compared to other structural elements. This increased deviation was likely due to the unique challenges of the manufacturing process, such as thermal gradients and cooling rates, which can cause more significant distortion in the vertical struts than in horizontal ones
M. Dallago et al. (2019) [77]	SLM (Strut based structures)	Examined how the number and severity of defects impact the mechanical properties.	Both the elastic modulus and fatigue resistance were strongly correlated with the number and severity of defects. Increased defects led to a lower elastic modulus and diminished fatigue resistance, indicating reduced material performance and reliability.
X. Y. Zhang et al. (2018) [129]	SLM (Strut based structures)	Examined the mechanical performance of as-built and as-heat-treated Functionally Graded Structures (FGSs) with pore sizes ranging from 350 to 450 μm.	Heat treatment substantially decreased the strength, with only a minor effect on the Young's modulus. The improvements in plasticity of the FGSs due to heat treatment were minimal.
O. Al-Ketan et al. (2018) [130]	Laser Beam PBF (both strut -based and TPMS structures)	To examine the discrepancy between the designed and printed relative densities, samples were weighed in air, and their actual relative density was calculated.	Each sample had a higher relative density than expected. The sheet-TPMS-based samples deviated the most from the CAD design, followed by the strut-based samples, whilst the solid-TPMS-based structures showed the smallest deviations.

## 5 Discussion

Designing and manufacturing porous implants presents a higher level of complexity compared to solid implants, primarily due to the need for precise control over pore architecture, which impacts both mechanical stability and biological integration. The development of porous implants involves intricate design considerations, advanced manufacturing techniques, and meticulous material selection to ensure optimal performance and biocompatibility. Additionally, the testing protocols for these implants are more demanding, requiring comprehensive evaluation of mechanical strength and biological responses. However, the advantages of porous implants, such as reducing stress shielding, enhancing osseointegration, extending longevity, and lowering the likelihood of secondary surgeries, outweigh these challenges. Addressing the complexities effectively necessitates the use of sophisticated design tools, innovative manufacturing technologies, and rigorous testing procedures, alongside strict adherence to regulatory standards. This multifaceted approach is crucial for realising the significant benefits that porous implants offer.

Although some additive-manufactured porous biomedical devices, such as cervical cages, have received FDA approval [131], it is noteworthy that no porous hip joint implant has yet obtained this approval for commercial use in orthopaedic treatments. The obstacles to obtaining FDA approval for a porous hip joint implant are multifaceted, involving the need for rigorous validation of biocompatibility, safety, and mechanical performance. The implant must demonstrate long-term stability, fatigue strength, and wear resistance under physiological conditions through extensive preclinical and clinical testing. Additionally, the manufacturing process must ensure consistent quality and performance, requiring detailed regulatory documentation. To streamline the approval procedure, early and frequent engagement with the FDA, utilisation of pre-submission programmes, leveraging existing data from similar approved devices, and employing innovative testing methods, such as computational modelling, are critical strategies. Collaborative regulatory science research can also advance understanding and create more efficient approval pathways, ultimately facilitating swifter introduction of these innovative implants to market. Consequently, further investigations are necessary to address the various aspects associated with replacing dense solid implants with porous structures.

The healthcare sector plays a crucial role in global sustainability, being responsible for 4.4 % of global greenhouse gas emissions and 10 % of the world's economic output [132]. The United Nations' Sustainable Development Goals (SDGs) highlight the link between healthcare and sustainability, emphasizing the need for the medical industry to adopt strategic, achievable goals to improve sustainability [133]. Additive manufacturing is crucial for improving sustainability in healthcare. Developing precise cost prediction models to compare AM with traditional manufacturing methods is complex and requires extensive knowledge and integration of design and production processes [134]. Traditional cost models favour conventional manufacturing for large-scale production, whilst AM is better for complex and customized items. AM supports lean production and streamlines supply chains, making it suitable for single-unit and low-volume manufacturing. Although AM machines require a higher initial investment, they are cost-effective for low volume runs and reduce material waste, enhancing resource efficiency [135]. Measuring AM costs is complex, however, most studies only concentrate on single-part production and often neglect broader supply chain impacts such as inventory, transportation costs, and disruption risks. Material costs significantly affect the overall expense of AM products. Adopting complementary technologies can enhance benefits and reduce raw material costs through economies of scale as AM use grows, which will potentially encourage further adoption [136]. AM offers several benefits, including lower supply chain costs, reduced lead times, less manpower, decreased energy consumption, minimised waste, and fewer tooling needs, along with enhanced efficiency, customization, and rapid production [137,138]. Neha et al. (2023) [133] found that AM reduces scaffold unit costs by 20 % compared to traditional manufacturing and uses about 17 % less energy. Whilst AM is more cost-effective for complex parts, CNC machining remains more economical for larger, simpler components. Monte Carlo sensitivity analysis indicates that processing and material costs are key factors for AM, highlighting the need for energy-efficient practices and circular design [139]. However, for large production runs, traditional manufacturing often remains more cost-effective than AM [137]. In summary, AM offers significant sustainability and efficiency benefits, particularly in low-volume, high-complexity production. Its cost-effectiveness and environmental advantages become more pronounced as adoption grows, potentially improving through economies of scale. This underscores the importance of ongoing research and strategic implementation in the healthcare sector.

The manufacturing process of porous implants begins with an in-depth analysis of the host bone. The primary objective is to create implant components that exhibit minimal disparity in mechanical stiffness compared to the surrounding bone. Consequently, it is crucial to assess the quality of the bone and estimate its stiffness and elastic modulus. These factors, which are integral to ensuring optimal implant performance, are detailed in the section on bone material properties. Another important aspect, that requires further study, would be to design a bespoke implant to provide the structural integrity necessary for various load conditions, from a large group of patients. This would entail exploring different approaches to mapping the design domain with porous structures, including the use of uniform, gradient, or other optimisation methods such as SIMP [23,86,94,115]. Additionally, manufacturing investigations of additively manufactured devices must be conducted, including suitable post-treatments [80,140,141] to address morphological deviations between the designed and manufactured models, as well as defects and their impact on the mechanical properties of the products [7,72,77,78,128]. Numerical analyses are a reliable tool for investigating critical regions, particularly in the context of failure and fatigue in hip implants. Established standards for implementing FEM in the design and analysis of these implants provide valuable guidelines. Additionally, mechanical tests, as specified in these standards (Table 5), are essential for validating the durability of the final implant design under physiological loads. These combined approaches will ensure that the implants meet performance and safety criteria.

Furthermore, the biological response of the porous implants needs to be assessed through in-vivo or in-vitro investigations, such as tribo-corrosion and biocompatibility [[142], [143], [144], [145], [146], [147], [148], [149], [150], [151], [152], [153]]. Comprehensive discussions are required to thoroughly examine the prospects of porous implants, ensuring confidence in their use within the human body. By addressing these diverse aspects, a clearer understanding of porous structures in hip joint implants can be achieved, facilitating their potential commercial utilisation for orthopaedic treatments.

## 6 Conclusion and future works

This comprehensive review paper has provided a focused analysis of porous hip joint implants, delving into three distinct sections: bone morphologies and biomechanical properties, solid hip joint implants and associated complications, and porous structures and porous implants. Examining these areas contributes to advancing knowledge and understanding in the field of hip joint implant design and development. The cortical bone of the femur, along with its thickness, has emerged as a critical determinant in ensuring the stability and longevity of hip joint implants. The mechanical properties and morphological characteristics of the femur bone, particularly its cortical region, play a pivotal role in selecting appropriate implant types and surgical techniques. In numerical studies, researchers have explored various mechanical behavior models, including complete isotropy, transverse isotropy, and anisotropy, to simulate and analyse the response of bone structures. However, thus far, no significant disparities have been observed when comparing the outcomes obtained from these distinct models. Solid hip joint implants, whilst offering higher stiffness upon implantation, have limitations. Complications such as stress shielding, cortical hypertrophy, and micromotion have been reported, underscoring the need for alternative solutions. Porous structures have emerged as a promising avenue for mitigating these challenges. By replacing dense solid materials with lattice structures, researchers have achieved significant advancements in addressing the limitations associated with solid implants.

The availability of diverse porous morphologies, each exhibiting unique mechanical behaviour, and the adoption of additive manufacturing techniques, have opened up new possibilities for tailoring implant designs to meet specific requirements, such as biocompatibility and mechanical compatibility with the surrounding bone tissue. A comprehensive investigation into the relationship between morphological parameters and the mechanical properties of different lattice structures is paramount. Notably, Triply Periodic Minimal Surfaces (TPMS) have garnered significant interest due to their average curvatures equal zero and smooth surfaces. These properties make TPMS structures particularly attractive from stress concentration and fatigue failure perspectives. Furthermore, the impact of porous morphologies on implant performance, including factors such as pore size, unit cell design, and interconnectivity, necessitates in-depth exploration to establish design guidelines for optimal implant performance. The theoretical aspects of porous implant design also warrants further investigation. Achieving an optimal distribution of porosity within the design domain, employing various topology optimisation methodologies, and developing strategies for seamlessly integrating lattice structures into the implant design process are among the critical theoretical challenges to address. In future studies, it is imperative to conduct comprehensive experimental investigations to validate the feasibility and performance of developed designs. This encompasses evaluating manufacturing defects, quantifying discrepancies between as-designed and as-built models, and the assessment of their impact on the mechanical behavior of porous implants. Furthermore, thorough examinations of biocompatibility, fatigue properties, fretting and tribo-corrosion phenomena, surface quality, and post-treatment procedures, such as surface finishing and heat treatment are essential areas of exploration. These multidisciplinary investigations will provide valuable insights, establishing a roadmap and benchmarking for implementing porous implants as a viable alternative to solid implants in orthopaedic applications.

Finally, this review paper has highlighted the potential of porous hip joint implants and emphasised the need for further research and development in this field. Addressing the challenges associated with solid implants and capitalising on the unique properties of porous structures can pave the way for improved implant designs. These advancements offer enhanced mechanical compatibility, reduced complications, and superior long-term performance.

## Data availability statement

No data was used for the research described in the article.

## CRediT authorship contribution statement

**Babak Ziaie:** Writing – original draft, Visualization, Validation, Resources, Methodology, Investigation, Formal analysis, Data curation, Conceptualization. **Xavier Velay:** Writing – review & editing, Visualization, Validation, Supervision, Project administration, Methodology, Conceptualization. **Waqas Saleem:** Writing – review & editing, Visualization, Validation, Supervision, Project administration, Methodology, Conceptualization.

## Declaration of competing interest

The authors declare that they have no known competing financial interests or personal relationships that could have appeared to influence the work reported in this paper.
